# Magnetic resonance-only rapid on-table planning and immediate treatment for spine metastases

**DOI:** 10.1016/j.phro.2025.100791

**Published:** 2025-06-11

**Authors:** Jerrold E. Kielbasa, Logan Kimble, Justin Rineer, Cameron W. Swanick, Patrick Kelly, Amish P. Shah

**Affiliations:** Department of Radiation Oncology, Orlando Health Cancer Institute, United States

**Keywords:** MR-guided radiotherapy, MRgRT, Palliative care, Spinal metastasis, Bone metastases, MR-only treatment planning

## Abstract

**Background and purpose:**

This work aims to develop a magnetic resonance (MR)-only treatment planning protocol for integration into magnetic resonance simulation (MR-SIM), on-table treatment planning, and immediate treatment workflow for rapid palliation of painful spine metastases.

**Materials and methods:**

Thirty-five treatment plans from healthy volunteers were generated on MR-SIM scans using a protocol including: (1) a library of 4 planning target volume (PTV) structures based on vertebral level, (2) default beam templates covering the PTVs while avoiding organs at risk (OAR), (3) bulk density assignments for dose calculations, and (4) a single set of dose optimization parameters. We transferred each plan to the patient’s prior computed tomography simulation (CT-SIM) images and compared dosimetric parameters. A time study was performed on the MR-SIM, on-table planning, and immediate treatment workflow for all 35 cases using healthy volunteers.

**Results:**

All bulk density MR-SIM plans resulted in acceptable dose distributions, with 100 % deemed appropriate for treatment by two physicians. The maximum point dose was within 3.2 %, and the minimum dose to 90 % of the target volume was within 2.8 % of the prescription dose. The time study demonstrated that the proposed workflow could be completed in a mean time of 23.6 min for the 3 Gy (10 fractions) plan and 25.4 min for the 8 Gy (single fraction) plan, from patient placement to treatment completion.

**Conclusion:**

These results demonstrate that safe, fast palliation of spine metastases can be achieved in under 30 min using MR-SIM, bulk density on-table planning, and immediate treatment delivery.

## 1 Introduction

As life-extending cancer treatments improve, an increased number of patients are living with metastatic disease [1]. Metastatic disease occurs frequently in the spine, commonly resulting in pain, spinal instability, pathological fracture, and spinal cord compression [2,3]. Radiation therapy is an effective tool for preventing and treating these conditions [2,3], but the conventional process from computed tomography simulation (CT-SIM) to treatment delivery can take several days to complete, leaving patients in considerable pain [4] and at risk for fracture or neurologic compromise, and the CT-SIM procedure itself can be difficult for patients to tolerate.

The need for more efficient treatment of spine metastases has led to proposed treatment methods that do not require traditional CT-SIM [5,[6], [7], [8], [9]]. Glober et al. showed that planning on diagnostic CT scans alone can be both safe and efficient [8]. Researchers at the University of Virginia developed a workflow (STAT RAD) in which the TomoTherapy (AccuRay Inc., Sunnyvale, CA) system is used to perform MVCT simulation, plan, and deliver dose in under 2 h [10]. Mittauer et al. introduced a MR-guided radiation therapy (MRgRT) workflow called STAT-ART (statum-adaptive radiotherapy), which utilizes pre-planning on previous diagnostic scans for treatment planning, then adapts that plan to the MR scan acquired with the patient on table [9]. The DART clinical trial further explored diagnostic scan-based radiation therapy, finding that this approach can significantly reduce patient time burden [11].

While diagnostic scan-based treatment planning is becoming an increasingly popular approach [12], not all patients have adequate diagnostic scans (whether CT or MRI) available for pre-treatment planning. The ViewRay MRIdian (ViewRay Inc., Oakwood Village, OH) offers a beneficial combination of features for imaging and treating palliative cases rapidly, including a magnetic resonance imaging (MRI) unit, a fully integrated adaptive treatment planning system (TPS), and a linear accelerator [13]. With MR-only treatment planning, the entire radiation therapy process (including simulation, treatment planning, IMRT quality assurance (QA), and dose delivery) could be performed without transitioning the patient off table and on the same days as consultation [[14], [15], [16],^17^]. This study was conducted in two parts: the first assessed the feasibility and dose accuracy of the proposed protocol using retrospective patient data, while the second explored the use of on-table MRI images from healthy volunteers to generate hypothetical palliative spine treatment plans. Here we present our workflow for rapid on-table treatment planning, dose-related analysis of the generated plans, and measured time of the entire process.

## 2 Materials and methods

### 2.1 Treatment planning protocol development

A standardized treatment planning protocol was developed using the ViewRay MRIdian treatment planning system (TPS) to enable rapid generation of radiotherapy plans for spine metastases based solely MR-SIM imaging. The protocol was designed to streamline planning while maintaining dosimetric accuracy and clinical feasibility. Key components included:

1. Default planning target volumes (PTVs) adaptable to patient anatomy (Fig. 1).

**Fig. 1:**
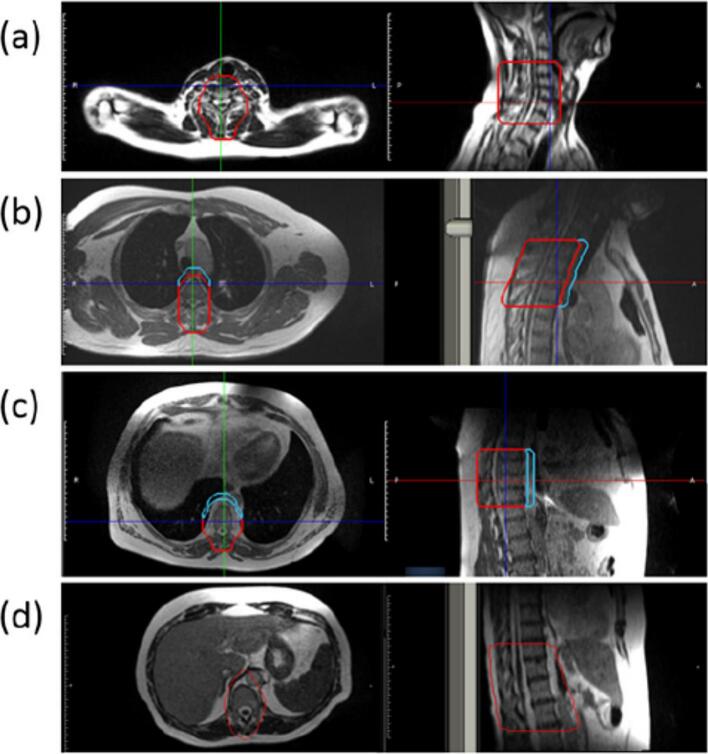
Default PTV structures (red) for the following vertebral ranges: (a) cervical spine (C3-C7), (b) superior thoracic spine (T1-T5), (c) mid-thoracic spine (T6-T11), and (d) inferior thoracic/lumbar spine (T11-L4). An anterior avoidance structure (blue) that is used to protect the esophagus at the thoracic level can be seen in (b) and (c). (For interpretation of the references to colour in this figure legend, the reader is referred to the web version of this article.)
2. Predefined beam templates for conformal PTV coverage and organ-at-risk (OAR) sparing.
3. Bulk tissue electron density assignments for MR-only dose calculation.
4. A single set of optimization parameters per prescription dose.

Detailed protocol specifications are provided in the Appendix*.* It is important to note that retrospective patient data sets were used to determine dose accuracy and the feasibility of the outlined protocol following IRB approval (IRB# 1998517-2).

#### 2.1.1 Target volumes and prescription doses

The protocol supports treatment of multiple spine levels, with two dose regimens: 30 Gy in 10 fractions and 8 Gy in 1 fraction. Four default PTVs were created to cover contiguous three-vertebrae regions in the thoracic and lumbar spine (T6–T11 and T12–L4). The default PTVs were adapted to each patient by deleting or extending the structure to fully cover the metastatic disease. For superior (T1–T5) and mid-thoracic (T6–T11) regions, anterior avoidance structures were used to spare the esophagus.

#### 2.1.2 Beam arrangements

**Fig. 2:**
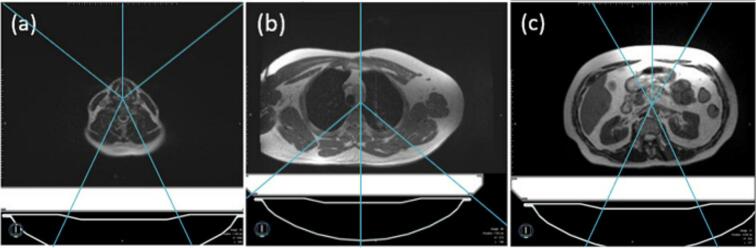
Default beam templates. (a) In the cervical spine region (C3-C7), a five-beam arrangement was used for uniform PTV coverage. (b) In both the superior thoracic (T1-T5) and mid-thoracic (T6-T11) spine regions, a four-beam template was used to spare lung dose. (c) In the inferior thoracic/lumbar spine region (T11-L4), a narrower five beam arrangement was used to spare kidney dose.

The beam templates for each of the four spine levels are shown in Fig. 2. Fig. 2a shows the five-beam arrangement used for cervical spine (C3-C7) plans. A four-beam arrangement was used at the superior thoracic spine (T1-T5) and mid-thoracic spine (T6-T11) levels to reduce lung dose (Fig. 2b). A five-beam arrangement was used to avoid the kidneys at the inferior thoracic/lumbar spine levels (T12-L4) (Fig. 2c).

#### 2.1.3 Image registration and contouring

For each patient, the default plan (PTV, beam arrangement, and bulk density assignment) most closely matching the treated spine level(s) was transferred to the MR-SIM dataset. Image registration was employed to ensure accurate targeting and precise patient alignment. Lung, heart, kidneys, and spinal canal were auto contoured and edited by a physician. Other OARs were not routinely contoured.^1^ Skin contours were auto generated by the TPS.

#### 2.1.4 Dose calculation

Bulk electron density assignments were applied to enable MR-only dose calculation using the following values: bone (vertebrae): ρ = 1.12, lung: ρ = 0.26, and water (all other tissues): ρ = 1.00. Only lung and vertebral structures required manual contouring for this purpose*.*

#### 2.1.5 Plan optimization and evaluation

IMRT plans were generated with the following objectives: ≥90 % of the PTV receiving the prescription dose, maximum point dose (D*_max_*) < 110 %, and minimization of dose to surrounding normal tissues. Plans were limited to 20 segments to facilitate efficient delivery. A plan was considered acceptable if ≥95 % of the prescription dose covered 90 % of the PTV, D*_max_* was <115 %, and all minimal OAR constraints were met. If these criteria were not achieved, the segment limit was increased to 25.

### 2.2 Retrospective dosimetric analysis of bulk density plans

To evaluate the accuracy of MR-only dose calculations, a retrospective analysis was performed using data from 35 patients previously treated on the MRIdian system. Following IRB approval (IRB# 1998517–2), each patient’s MR-SIM and CT-SIM datasets were imported into the ViewRay TPS and rigidly fused.

Using the MR-only protocol from Section 2.1, 70 simulated spine plans were created: 35 for 30 Gy in 10 fractions and 35 for 8 Gy in 1 fraction. Each MR-based plan was then transferred to the corresponding CT dataset and recalculated using CT-derived densities. Fig. 4 shows representative plans for each treatment region and resulting dose distribution. Dosimetric comparison included: minimum dose to 90 % of the PTV (*D_90_*) and the maximum dose point (*D_max_*). A plan was considered acceptable if the dose to 90 % of the volume was greater than 95 % of the prescription dose and the minimally acceptable constraints listed above were met. OAR dose metrics included: (1) esophageal D_max_, (2) lung volume receiving 10 Gy/7.4 Gy (for *D*_Rx_ = 30 Gy/8Gy), (3) heart volume receiving 15 Gy/8.6 Gy, and (4) kidney volume receiving 15 Gy/8.6 Gy.

### 2.3 Clinical workflow

**Fig. 3:**
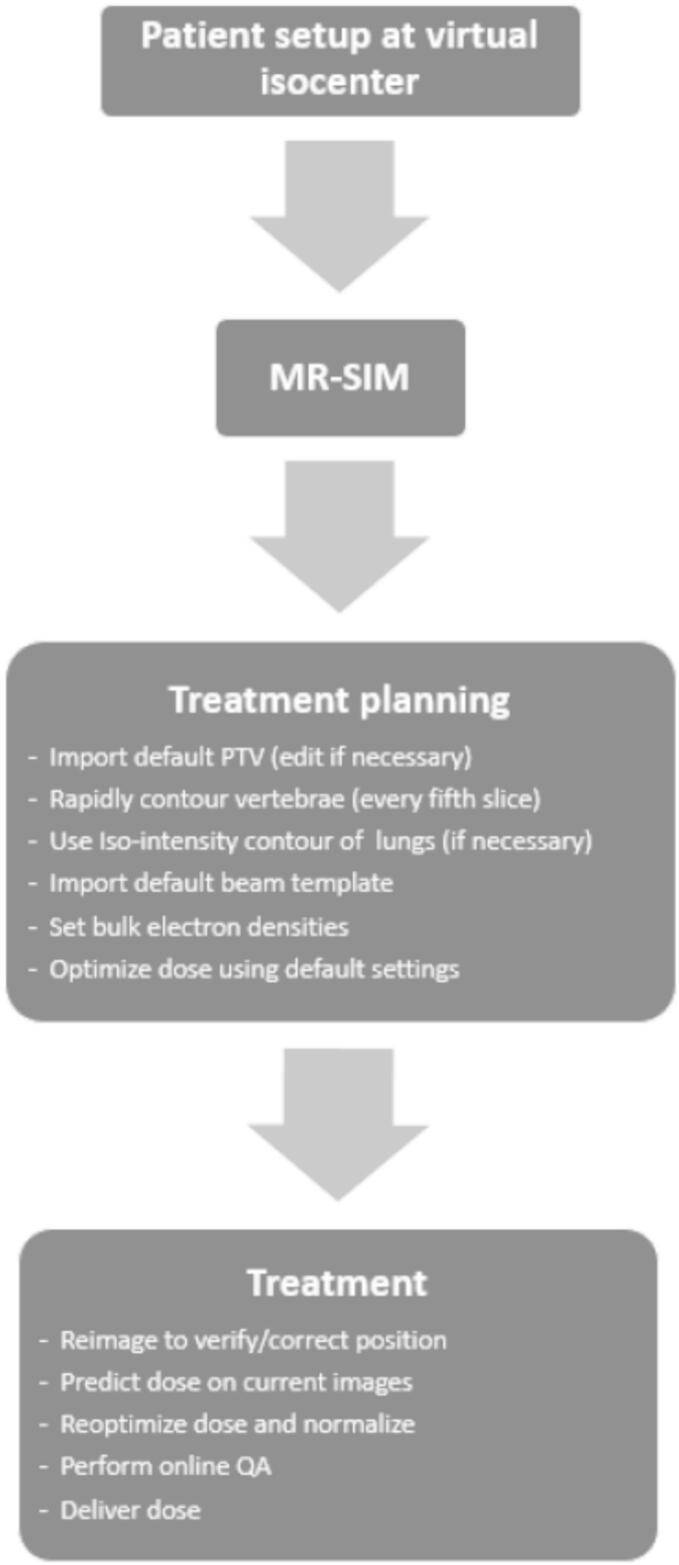
Proposed workflow for MR-only on-table planning and immediate treatment.

A clinical workflow was developed to enable MR-only, on-table planning with immediate treatment delivery (Fig. 3). Full details are included in the Appendix.

**Fig. 4:**
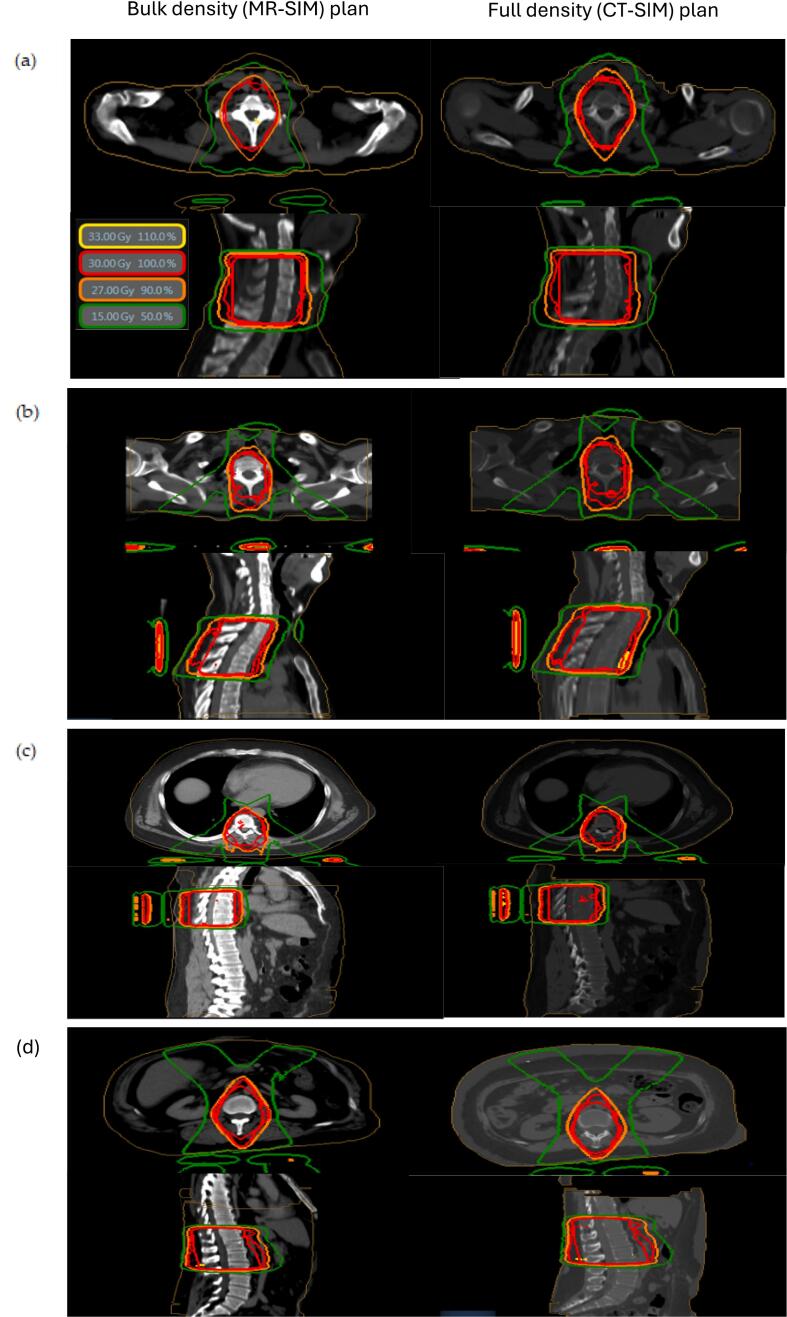
Example dose distributions for (left) bulk density MR-SIM plans and (right) the resulting dose distribution when that plan is transferred to the patient’s full density CT-SIM scan. Plans were generated using the protocols for the (a) cervical spine (C3-C7), (b) superior thoracic spine (T1-T5), (c) mid-thoracic spine (T6-T11), and (d) the inferior thoracic/ lumbar spine (T11-L4) regions.

Briefly, the therapist initially positions the patient near the virtual isocenter^†^^2^. The patient is then moved into the bore for MR-SIM acquisition. Following imaging, the patient may be removed from the bore for comfort while the plan is generated using the MR-only protocol.

If approved by the physician, the patient is repositioned at isocenter and reimaged. Couch shifts are calculated and applied. A new adapted plan is generated using weight optimization and renormalization. The ViewRay online QA tool performs a secondary dose calculation check.

After QA review, cine MR is used for target tracking, and treatment is delivered.

### 2.4 Clinical workflow time study with healthy volunteers

To test the clinical workflow, after institutional review board approval (IRB# 1254739-8), we performed 10 simulated treatments on health volunteers. Healthy volunteers were used to avoid causing discomfort to patients with spinal metastasis while assessing our workflow. The following times were recorded: (1) setup, (2) MR simulation, (3) treatment planning, (4) reimaging and couch shifts, and (5) dose prediction, re-planning, and plan QA. Volunteers were then removed from the table without dose delivery and ViewRay TPS predicted treatment time for each plan. The sum of these times was recorded as the total time that elapses from initial patient positioning to treatment completion.

## 3 Results

### 3.1 Dose-related analysis of bulk density plans

The proposed bulk density treatment planning protocol was tested on 35 cases, each with a 3 Gy (10 fraction) and an 8 Gy (single fraction) plan. Each plan was optimized independently and is therefore unique. The results of the dose-related analysis study are presented in Table 1. The mean and range of each dosimetric quantity is shown for both the bulk density MR-SIM plans as well as for the dose distributions resulting from the transfer of each plan to the patient’s full density CT-SIM scan. The maximum absolute percent variation of the full density CT-SIM dose metric from the bulk density MR-SIM value is shown in the last column, with the largest variation in *D*_max_ being 3.2 %, and the largest variation in *D*_90_ being 2.8 %. A plan was considered acceptable if the dose to 90 % of the volume was greater than 95 % of the prescription dose and the minimally acceptable constraints listed above were met.

**Table 1:** Summary of dosimetric parameters for bulk density plans and corresponding data resulting from transfer of the plans to full density CT-SIM scans.

Vertebral level	*D*_rx_	Parameter (units)		Bulk density (MR-SIM)		Full-density (CT-SIM)		Max absolute % variation from bulk density value
				Mean	Range	Mean	Range	
Cervical spine (C3-C7)	30 Gy	*D*_max_	(Gy)	33.2	32.9–33.5	33.1	32.7–33.5	1.0
		*D*_90_	(Gy)	30.0	*normalized*	30.3	30.1–30.5	1.6
		*D*_max,esophagus_	(Gy)	31.1	26.2–32.5	31.0	26.6–32.3	1.5
	8 Gy	*D*_max_	(Gy)	8.9	8.7–9.0	8.8	8.7–8.9	1.8
		*D*_90_	(Gy)	8.0	*normalized*	8.1	8.0–8.1	1.6
		*D*_max,esophagus_	(Gy)	8.4	7.4–8.8	8.4	7.4–8.9	0.5

Superior thoracic spine (T1-T5)	30 Gy	*D*_max_	(Gy)	33.2	32.5–33.9	33.4	32.5–34.2	2.5
		*D*_90_	(Gy)	30.0	*normalized*	29.9	29.6–30.1	1.3
		*D*_max,esophagus_	(Gy)	31.7	30.7–33.6	32.2	30.6–33.8	3.2
		*V*_10Gy,lung_	(%)	8.6	5.5–14.1	9.2	6.0–14.5	11.3
	8 Gy	*D*_max_	(Gy)	8.9	8.7–9.1	8.9	8.7–9.2	1.6
		*D*_90_	(Gy)	8.0	*normalized*	8.0	7.9–8.0	1.0
		*D*_max,esophagus_	(Gy)	8.4	8.2–8.7	8.5	8.2–8.7	3.4
		*V*_7.4Gy,lung_	(cm^3^)	59.5	10.9–343.0	56.8	7.6–363.6	51.4

Mid-thoracic spine (T6-T11)	30 Gy	*D*_max_	(Gy)	33.3	32.8–33.8	33.8	32.8–34.4	2.4
		*D*_90_	(Gy)	30.0	*normalized*	29.9	29.7–30.1	1.2
		*D*_max,esophagus_	(Gy)	27.9	11.8–32.0	28.1	11.9–32.6	2.9
		*V*_10Gy,lung_	(%)	12.1	6.7–18.5	12.7	7.0–18.9	8.1
		*V*_15Gy,heart_	(%)	8.1	0.0–22.9	8.5	0.0–24.0	13.7
	8 Gy	*D*_max_	(Gy)	8.9	8.8–9.2	9.0	8.9–9.1	2.3
		*D*_90_	(Gy)	8.0	*normalized*	8.0	7.9–8.0	1.4
		*D*_max,esophagus_	(Gy)	7.5	3.3–8.7	7.6	3.3–8.8	3.0
		*V*_7.4Gy,lung_	(cm^3^)	26.3	0.6–51.6	22.1	0.4–55.0	35.6
		*V*_8.6Gy,heart_	(cm^3^)	0.0	0.0–0.0	0.0	0.0–0.0	−

Inferior thoracic/lumbar spine (T11-L4)	30 Gy	*D*_max_	(Gy)	33.3	32.7–33.8	33.4	33.1–33.7	3.2
		*D*_90_	(Gy)	30.0	*normalized*	30.0	29.7–30.8	2.8
		*V*_15Gy,kidney_	(%)	6.5	0.0–12.7	6.5	0.0–12.1	6.4
	8 Gy	*D*_max_	(Gy)	8.9	8.8–9.1	8.9	8.8–9.1	1.4
		*D*_90_	(Gy)	8.0	*normalized*	8.0	7.9–8.1	1.4
		*V*_8.6Gy,kidney_	(cm^3^)	0.0	0.0–0.0	0.0	0.0–0.0	−

*Abbreviations*: *D*_Rx_ = prescription dose; *D*_max_ = maximum dose in the treatment plan; *D*_90_ = dose covering 90 % of the planning target volume; *D*_max, esophagus_ = maximum dose to the esophagus; *V*_[X]Gy, [organ]_ = Volume of [organ] receiving [X] Gy; MR-SIM = magnetic resonance simulation; CT-SIM = computed tomography simulation.

OAR avoidance was accomplished with strategically designed beam angles (Fig. 2). Beam angles alone did not provide adequate protection to the OAR at the mid-thoracic level, the esophagus, so a 1-cm avoidance structure was added to the anterior of the PTV of the superior and mid-thoracic spine, effectively driving down the dose. The maximum dose to the esophagus for bulk density plans was within 3.4 % of the *D_max_* of the full density plans. With the 3 Gy plans, the maximum volume for $V_{15Gy,\;\;kidney}^{\mathrm{MR}-SIM}$ was 12.7 %, and the maximum volume for $V_{15Gy,\;kidney}^{\mathrm{CT}-SIM}$ was 12.1 %. In the 8 Gy plans, the maximum $V_{7.4Gy,\;lung}^{\mathrm{MR}-SIM}$ was 343.0 cc, and the maximum $V_{7.4Gy,\;lung}^{\mathrm{CT}-SIM}$ was 363.6 cc. No volume of the heart or kidney received 8.6 Gy. When compared to AP/PA or 3D conformal plans, the current standard of care in urgent palliative spinal metastasis treatment, the IMRT plans generated in this study result in superior dose distribution and OAR avoidance [18].

### 3.2 Time study

Table 2 summarizes the time study results. The mean times for each step measured with the volunteers were (1) 4.4 min for patient set-up, (2) 1.5 min for MR-SIM, (3) 8.2 min for planning, (4) 2.8 min for reimaging and positioning, (5) 2.7 min for dose prediction, plan reoptimization, and online QA, and (6) 4.1 min for predicted 3 Gy treatment delivery (5.9 min for predicted 8 Gy treatment delivery). The total mean time to treat a 3 Gy or 8 Gy fraction would be 23.6 min or 25.4 min, respectively. The patient would need to remain completely still for MR-SIM, re-imaging in the bore, and treatment time, which equate to 8.4 ± 1.1 and 10.2 ± 1.3 for 3 Gy and 8 Gy, respectively. These durations are comparable to times required during traditional CT-SIM, with no need for the patient to transfer off table.

**Table 2:** Results of the time study of the proposed workflow for MR-only simulation, on-table planning, and immediate treatment. Treatment time was predicted by the treatment planning system.

Mean ± standard deviation (min)	
Patient setup	4.4 ± 1.1
MR-SIM	1.5 ± 0.1
Treatment planning	8.2 ± 1.9
Reimage and shift	2.8 ± 0.6
Predict dose, weight optimization, and online IMRT QA	2.7 ± 0.5
Treatment time *predicted* (3 Gy × 10/8 Gy × 1)	4.1 ± 0.4/5.9 ± 0.6

Total (3 Gy × 10/8 Gy × 1)	23.6 ± 2.5/25.4 ± 3.0

*Abbreviations*: MR-SIM = magnestic resonance simulation; IMRT = intesity modulated radiation therapy; QA = quality assurance.

## 4 Discussion

Prompt initiation of palliative radiation therapy is critical for relieving pain and improving quality of life in patients with metastatic cancer. Many institutions have adopted expedited treatment workflows using diagnostic scans in place of CT simulation [[6], [7], [8], [9],^11^]. However, this approach is limited when diagnostic scans are unavailable. To address this gap, we developed an MR-only planning protocol using the ViewRay MRIdian system that enables same-day palliative spine treatment without the need for prior imaging.

Using healthy volunteers, we demonstrated that this protocol can reliably generate and deliver treatment plans for spinal lesions from C3 to L4 in under 30 min—supporting streamlined, same-day care. Unlike pre-planning workflows that rely on previously acquired diagnostic scans, our protocol uses a single MR-SIM scan acquired immediately after patient setup. All treatment steps—imaging, planning, and delivery—occur with the patient in position. This not only simplifies logistics but also reduces patient discomfort and travel, potentially improving quality of life.

The primary challenge in MR-only planning is electron density assignment. While bulk density overrides introduce some uncertainty, prior studies by Jonsson et al. [16] and Lambert et al. [15] have shown that this approach yields clinically acceptable dose distributions. In our study, using three bulk densities—water, bone, and lung—produced accurate palliative treatment plans, with a D_90_ variance of 2.8 % and a maximum dose (D*_max_*) error under 3.2 %. Notably, Jonsson et al. [19] reported a maximum MU calculation error of 1.6 % using bulk densities in brain, thoracic, and pelvic plans—without accounting for bone. Our inclusion of bone density further improves accuracy, particularly in spine cases. Additionally, MR-only same-day treatment remains rare and can strain clinical resources or increase the risk of workflow errors [20]. To mitigate this, our protocol aligns with the existing adaptive framework of the MRIdian system but removes the need for prior imaging (Fig. 4). Unlike the STAT-ART protocol by Mittauer et al., which requires CT pre-planning and deformable registration, our method completes all steps during a single visit [9].

Schiff et al. developed the MRI-guided Adaptive Palliative Radiotherapy (MAP-RT) protocol, treating 16 patients without prior CT scans using MR-only planning and bulk density overrides [12]. However, MAP-RT separated planning and treatment sessions, with a median time of 407 min between simulation and delivery. Their median treatment duration was 41 min, while our workflow completed the full process—from setup to treatment—in approximately 30 min.

MacDonald et al. demonstrated a CT-free workflow using the Varian Ethos platform and an anthropomorphic phantom [21]. They used rapid cone beam CT (CBCT) for spine and prostate treatments, completing spine workflow in 33 min with <2 % deviation between planned and delivered dose. However, using a phantom limited assessment of real-world variability.

Similarly, Nelissen et al. introduced the FAST-METS CBCT workflow on Ethos, which required a prior diagnostic CT for pre-planning [22]. Patients were pre-screened via virtual consultation and treated during a single clinic visit. Their average treatment time of 30 min was comparable to ours. However, unlike our protocol, FAST-METS relied on pre-generated plans adapted on treatment day. In contrast, our entire process—from planning to delivery—is performed with the patient on the treatment table.

The MRIdian and Ethos platforms represent two distinct approaches to adaptive radiotherapy. In the standard adaptive workflows, MRIdian provides superior soft tissue contrast and intrafraction motion tracking via real-time MRI without additional imaging dose—key advantages in the palliative setting. While real-time target tracking is not strictly necessary for palliative treatments, it does provide an additional safeguard, especially in the 8 Gy × 1 fraction regime. Ethos, while CT-based, offers rapid plan adaptation using daily CBCT, though its limited soft tissue contrast may affect accuracy [23]. Both platforms support the development of efficient same-day workflows.

This study has several limitations. First, our time study was conducted on healthy volunteers rather than patients with metastatic cancer, who may have reduced mobility or tolerance for prolonged positioning. Thus, the recorded times may underestimate actual clinical durations. However, the protocol itself is unchanged, and the single setup minimizes repositioning and patient movement. Our predicted beam-on times—4.1 min for 3 Gy and 5.9 min for 8 Gy—are comparable to conventional treatments. Second, bone infiltration in patients with spinal metastases may affect local tissue density and dosimetric accuracy.^3^ However, given the small difference between the assigned electron densities of soft tissue (ρ = 1.00) and bone (ρ = 1.12), this impact is likely minimal [19]. Finally, a broader limitation is the limited availability of MR-guided systems like MRIdian. These systems are expensive and require specialized training in MR-based planning and adaptive workflows. As availability grows, this protocol can be more widely implemented. Although initially designed for palliative spine cases, the workflow is now being adapted for more complex palliative and select definitive treatments where real-time imaging and adaptive planning offer clinical advantages.

This study demonstrates that same-day, MR-only palliative spine radiotherapy can be safely and efficiently delivered in under 30 min using the MRIdian system. By incorporating bulk electron densities, accurate treatment plans can be generated without prior CT simulation. The workflow aligns with existing MRIdian practices and can be readily adopted in clinical settings, offering a fast, patient-centered approach to high-quality palliative care.

## Institutional review board statement

The study was conducted in accordance with the Declaration of Helsinki and approved by the Institutional Review Board of Orlando Health.

## Author contributions

All authors have read and agreed to the published version of the manuscript. P.K., J.R., A.P.S, and J.E.K were responsible for the conceptualization of the project. P.K. and A.P.S. were responsible for funding acquisition. J.E.K., R.M., and A.P.S. were responsible for methodology and data curation. P.K., A.P.S., and J.E.K performed the formal analysis. J.E.K and L.K. prepared the original draft of the manuscript. All authors were involved in reviewing and editing the manuscript. 

## Informed consent statement

Informed consent was obtained from all subjects involved in the study.

## Data availability statement

Research data are not available at this time.

## Funding

This research was funded by ViewRay Inc (grant ID: 00001-28001-7192). (Oakwood Village, OH, U.S.A).

## Declaration of competing interest

The authors declare the following financial interests/personal relationships which may be considered as potential competing interests: P.K. received grant support from ViewRay for this project and Varian Medical System. A.P.S. has received grant support and honoraria from ViewRay and Varian Medical Systems. He has also received consulting fees from ViewRay. J.E.K., L.C.K., and J.K. have no conflicts to disclose. The funders had no role in the design of the study; in the collection, analyses, or interpretation of data; in the writing of the manuscript, or in the decision to publish the results. 

## Footnotes

1 Consistent with current palliative treatment of painful spine metastases, OARs are not contoured in this protocol. These were, however, contoured as part of this study for dosimetric evaluation to demonstrate the efficacy of the protocol at limiting dose to them.

2 While target tracking is not strictly necessary for treatment of spinal metastasis due to lack of organ motion, it is part of the normal workflow for the ViewRay MRIdian, and there is no reason not to utilize this function.

3 However, the MRIdian uses the TrueFISP sequence, for which bone is easily identifiable.
