# Supplementary material for: Magnetic resonance-only rapid on-table planning and immediate treatment for spine metastases
Source: Phys Imaging Radiat Oncol. 2025 Jun 11;35:100791. doi: 10.1016/j.phro.2025.100791 (PMC12226384; doi:10.1016/j.phro.2025.100791)
Supplement: Supplementary Data 1 [file mmc1.docx]

**Appendix A**

*A.1 Detailed Treatment Planning Protocol*

Contours

The ViewRay treatment planning system (TPS) allows users to save structure templates that streamline contouring. To accommodate the variations in curvature and vertebral size along the spine, four PTVs were developed to cover approximately three vertebrae in the following regions: cervical spine (C3-C7), superior thoracic spine (T1-T5), mid-thoracic spine (T6-T11), and inferior thoracic spine/lumbar spine (T11-L4). When loading the structure template for treatment planning, the selected PTV is manually registered to the patient’s disease site. Examples of each of these structures applied to patient MR-SIM scans are shown in Figure 1. Extension or shortening of the PTV along the craniocaudal direction can be easily done in the TPS using either the copy/paste or delete functions. In cases where the default PTV does not appropriately correspond to the patient’s anatomy, manual editing of the structure can be done quickly in the TPS. For the superior thoracic (T1-T5) and mid-thoracic (T6-T11) spine regions, an additional dose optimization structure is used to prevent hot spots in the esophagus (Figs. 1b and 1c). This avoidance structure is generated by expanding the PTV by 1 cm in the anterior direction and then using the Boolean function to subtract the PTV from it.

The vertebrae and lungs are the only remaining structures necessary for this protocol. The vertebrae must be contoured manually; this is done rapidly by (1) limiting the extent of the structure to three slices superior and three slices inferior to the PTV and (2) only contouring every fifth slice and using the interpolation tool to fill in the gaps. The lung contour is generated using the iso-intensity tool in the TPS.

Beams

The beam arrangements for each of the vertebral levels were designed to balance OAR avoidance and the uniformity of PTV coverage. For the cervical spine plans, beams are placed at 0°, 52°, 155°, 205°, and 308° (Figure 2a). For treatments at both the superior thoracic spine (T1-T5) and mid-thoracic spine (T6-T11) levels, lung dose was reduced by placing beams at 0°, 130°, 180°, and 230° (Figure 2b) to reduce lung dose. At the inferior thoracic spine/lumbar spine level (T11-L4), beams were placed at 0°, 30°, 155°, 205°, and 330° (Figure 2c) to minimize the dose to the kidneys.

Dose

The most pertinent dose calculation and optimization parameters are shown in Table A1. The treatment plans are limited to 20 segments to keep the treatment time short. If a 20-segment plan does not produce acceptable results, five segments are added. The objectives used were found to produce acceptable treatment plans both reliably and quickly. The skin structure is set to decrease at 15 Gy/4 Gy for 30 Gy/8 Gy prescriptions. The PTV is set to increase at 32 Gy/8.5 Gy and decrease at 30 Gy/8 Gy. While ViewRay discourages conflicting objectives, we found that this combination consistently resulted in better plans that could be delivered with fewer than 25 segments. The anterior avoidance structure, which is only used for superior thoracic (T1-T5) and mid-thoracic (T6-T11), is set to decrease at 27 Gy/7.2 Gy. The bulk electron densities relative to water (*ρ*) used for dose calculation are *ρ*_bone_ = 1.12 (assigned to vertebrae), *ρ*_lung_ = 0.26 (assigned to lung), and *ρ*_water_ = 1.00 (assigned to all remaining tissue). The resulting plan dose is normalized so that 90% of the PTV would receive the prescription
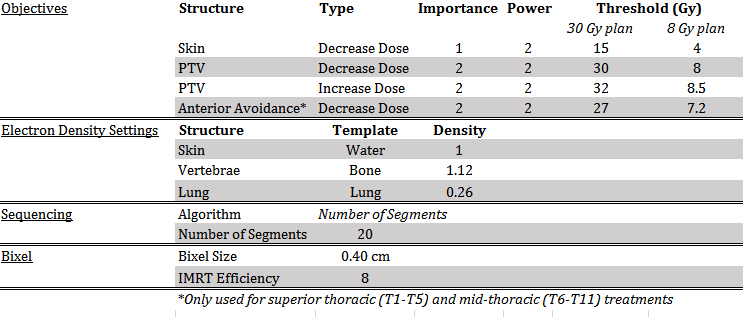
dose.

*Abbreviations:* PTV = planning target volume; IMRT = Intensity modulated radiation therapy.

**Table A1** Summary of dose calculation and optimization parameters used for bulk density treatment plans

**Table A2** Acceptable plan metrics for OARs

| D_rx_ = 30 Gy | D_rx_ = 8 Gy |
| --- | --- |
| *V_27 Gy, lung_* < 1000 cc | *V_7.4 Gy, lung_* < 1000 cc |
| *V_33 Gy, heart_* < 2 cc | *V_8.6 Gy, heart_* < 2 cc |
| *V_15 Gy, kidney_* < 30% | *V_8.6 Gy, kidney_* < 2 cc |

*A.2 Detailed Clinical Workflow*

The clinical workflow (Figure 4) was developed to streamline the implementation of MR-only on-table planning and immediate treatment. In this workflow, contouring templates and beam templates are used to decrease on-line planning time. The four contouring templates each contain one of the default vertebral PTVs (Figure 1) and empty bone and lung structures (the superior thoracic (T1-T5) and mid-thoracic (T6-T11) templates also include an empty avoidance structure to protect the esophagus from hotspots). Three beam templates are used: A five-beam template (Figure 2a) for the cervical spine (C3-C7), a four-beam template (Figure 2b) for the superior thoracic (T1-T5) and mid-thoracic (T6-T11) spine, and a five-beam template (Figure 2c) for the inferior thoracic/lumbar (T11-L4) spine. Prior to patient set up, both the diagnosis and prescription must be completed.

MR-only Simulation

The patient is positioned with his/her arms down, and the therapist approximates the midline, vertical, and superior-inferior positioning at the virtual isocenter of the ViewRay MRIdian. The patient is then sent into the bore to the treatment/imaging isocenter, and a 25 sec MR-SIM scan is acquired (54 cm × 47 cm × 25 cm field of view). Once the scan is complete, and the patient’s position is determined to be adequate for treatment, the couch coordinates are recorded. At this point the patient can be sent out of the bore for comfort while on-table treatment planning is completed.

On-table Treatment Planning

The MR-SIM scan is selected for contouring. The ViewRay TPS automatically contours the skin. The appropriate contour template is loaded and the PTV is registered to the disease site. If the PTV does not adequately conform to the disease site, it can be quickly edited with the nudge tool in the axial planes and the copy/paste/delete functions in the superior-inferior direction. If the disease site is in the superior thoracic (T1-T5) or mid-thoracic region (T6-T11), an avoidance structure anterior to the PTV must be generated to protect the esophagus. This is done by extending the PTV 1cm in the anterior direction and then subtracting off the PTV. The bone structure is then generated by rapidly contouring the vertebrae on every fifth slice starting at three slices superior to the PTV and ending at three slices inferior to the PTV (or vice versa). If lung tissue is in the beam path, the lung contour is generated using the ViewRay TPS iso-intensity contour function. The resulting contour will have islands and holes that can be removed using the clean-up tool. These are the only contours necessary for this protocol.

After the contouring is completed and approved, the couch coordinates that were recorded during simulation are entered. Next the appropriate beam template is loaded. At this point the dose optimization can be run. The details are discussed in section S.1, and the parameters can be found in Table A1.

Repositioning, Reimaging, Online QA, and Treatment

If the physician considers the resulting treatment plan acceptable, the plan can be loaded for delivery. At this point the patient can be returned to isocenter. The patient is reimaged with a 17 sec scan (45 cm × 45 cm × 24 cm field of view). Fusing this scan with the MR-SIM scan will determine what couch shifts, if any, are needed. Once the shifts are sent to the couch, the original plan dose is predicted on the current image set. A new plan is generated by weight optimization and renormalization ($D_{90\%}^{MR-SIM}$ = *D*_Rx_). It is critical to generate this new plan because it is required for the use of ViewRay’s online QA tool. Once the plan passes the QA, cine images are acquired for target tracking and the treatment is delivered.
